# The NUHM2 after LHC Run 1

**DOI:** 10.1140/epjc/s10052-014-3212-9

**Published:** 2014-12-17

**Authors:** O. Buchmueller, R. Cavanaugh, M. Citron, A. De Roeck, M. J. Dolan, J. R. Ellis, H. Flächer, S. Heinemeyer, S. Malik, J. Marrouche, D. Martínez Santos, K. A. Olive, K. J. de Vries, G. Weiglein

**Affiliations:** 1 High Energy Physics Group, Blackett Laboratory, Imperial College, Prince Consort Road, London, SW7 2AZ UK; 2 Fermi National Accelerator Laboratory, P.O. Box 500, Batavia, IL 60510 USA; 3 Physics Department, University of Illinois at Chicago, Chicago, IL 60607-7059 USA; 4 Physics Department, CERN, 1211 Geneva 23, Switzerland; 5 Antwerp University, 2610 Wilrijk, Belgium; 6 Theory Group, SLAC National Accelerator Laboratory, 2575 Sand Hill Road, Menlo Park, CA 94025-7090 USA; 7 Theoretical Particle Physics and Cosmology Group, Department of Physics, King’s College London, London, WC2R 2LS UK; 8 H.H. Wills Physics Laboratory, University of Bristol, Tyndall Avenue, Bristol, BS8 1TL UK; 9 Instituto de Física de Cantabria (CSIC-UC), 39005 Santander, Spain; 10 NIKHEF and VU University Amsterdam, Science Park 105, 1098 XG Amsterdam, The Netherlands; 11 William I. Fine Theoretical Physics Institute, School of Physics and Astronomy, University of Minnesota, Minneapolis, MN 55455 USA; 12 DESY, Notkestraße 85, 22607 Hamburg, Germany

## Abstract

We make a frequentist analysis of the parameter space of the NUHM2, in which the soft supersymmetry (SUSY)-breaking contributions to the masses of the two Higgs multiplets, $m^2_{H_{u,d}}$, vary independently from the universal soft SUSY-breaking contributions $m^2_0$ to the masses of squarks and sleptons. Our analysis uses the MultiNest sampling algorithm with over $4 \times 10^8$ points to sample the NUHM2 parameter space. It includes the ATLAS and CMS Higgs mass measurements as well as the ATLAS search for supersymmetric jets + ${/\!\!E}_T$ signals using the full LHC Run 1 data, the measurements of $\mathrm{BR}(B_s \rightarrow \mu ^+\mu ^-)$ by LHCb and CMS together with other B-physics observables, electroweak precision observables and the XENON100 and LUX searches for spin-independent dark-matter scattering. We find that the preferred regions of the NUHM2 parameter space have negative SUSY-breaking scalar masses squared at the GUT scale for squarks and sleptons, $m_0^2 < 0$, as well as $m^2_{H_u} < m^2_{H_d} < 0$. The tension present in the CMSSM and NUHM1 between the supersymmetric interpretation of $(g-2)_\mu$ and the absence to date of SUSY at the LHC is not significantly alleviated in the NUHM2. We find that the minimum $\chi ^2 = 32.5$ with 21 degrees of freedom (dof) in the NUHM2, to be compared with $\chi ^2/\mathrm{dof} = 35.0/23$ in the CMSSM, and $\chi ^2/\mathrm{dof} = 32.7/22$ in the NUHM1. We find that the one-dimensional likelihood functions for sparticle masses and other observables are similar to those found previously in the CMSSM and NUHM1.

## Introduction

Supersymmetric (SUSY) models are among the best-motivated extensions of the Standard Model (SM) that might be discovered at the Large Hadron Collider (LHC). They stabilise the electroweak hierarchy [1, 2] and facilitate grand unification [3–7], and the lightest supersymmetric particle (LSP) provides a natural candidate for the cosmological dark matter [8, 9]. However, the absence of a signal in direct searches for SUSY particles in Run 1 of the LHC [10, 11] sets strong constraints on supersymmetric models, as do the measurement of the mass and properties of the Higgs boson [12, 13] and precision measurements of rare decays such as $B_s \rightarrow \mu ^+ \mu ^-$ [14–16].

Gaining a fully accurate picture of the effects of these constraints requires that they be combined in global statistical fits within specific supersymmetric models. Particularly well-motivated and simplified versions of the minimal supersymmetric Standard Model (MSSM) [17, 18] are derived from grand unified theory (GUT) model-building considerations. There have been a number of analyses [19–42] of the constraints imposed by LHC Run 1 data on the parameter spaces of such models, particularly the constrained MSSM (CMSSM) [43–60], whose parameters are the soft supersymmetry (SUSY)-breaking masses $m_0$, $m_{1/2}$ and $A_0$ that are universal at the GUT scale, and $\tan \beta$, the ratio of the two vacuum expectation values of the two Higgs doublets. There have also been some studies of the LHC constraints on the NUHM1 [61–64], in which the soft SUSY-breaking contributions to the masses of the electroweak Higgs multiplets, $m^2_{H_d, H_u}$, are equal but non-universal.

However, these models have become very constrained by the recent data. The anomalous magnetic moment of the muon $(g-2)_\mu$ [65–74] is a particular source of tension, as has been reinforced by the recent convergence in the Standard Model (SM) calculations of $(g-2)_\mu$ based on $\tau$ decays and different sets of $e^+ e^-$ annihilation data [75, 76]. As is well known, the ${\sim }3.5\,\sigma$ discrepancy between the observed value and SM prediction can be reduced by SUSY contributions due to relatively light electroweakly interacting superpartners. In the simple GUT-based models mentioned above, direct searches and the Higgs mass force the coloured super-partners to be so heavy that, due to the universality of the soft SUSY-breaking parameters $m_0$ and $m_{1/2}$ at the GUT scale that leads also to relatively heavy electroweak superpartners, these models cannot remove the $(g-2)_\mu$  anomaly [77]. Also for this reason, the LHC searches for leptons and electroweak inos do not impact significantly the parameter spaces of these GUT-based models.

A related extension of these models which a priori might be able to alleviate this tension is the NUHM2 [78, 79], in which $m^2_{H_d} \ne m^2_{H_u} \ne m_0^2$ in general,^1^ but the soft SUSY-breaking parameters $m_0$, $m_{1/2}$ and $A_0$ are still universal at the GUT scale. An equivalent formulation of the NUHM2 is to treat the pseudoscalar mass $M_A$ and supersymmetric Higgs mass term $\mu$ as free parameters, which could lead to interesting phenomenology associated with light higgsinos and/or a light pseudoscalar Higgs. Moreover, new terms in the renormalisation group equations (RGEs) associated with the scalar-mass non-universality in the NUHM2 may lead to lighter left-handed sleptons, offering further avenues for ameliorating the tension with $(g-2)_\mu$ (see Sect. 3.1 for details).

Therefore, in this paper we extend our previous analyses of the CMSSM and NUHM1 [19] to the NUHM2 [78, 79], and compare the corresponding phenomenological predictions. In addition to the 8 TeV ATLAS search for supersymmetry in the jets + ${/\!\!E}_T$ channel [10]^2^ channel, our frequentist fit using the MultiNest [80] sampling algorithm includes Higgs mass measurements [12, 13], the measurements of $\mathrm{BR}(B_s \rightarrow \mu ^+\mu ^-)$ by LHCb and CMS [14–16], other B-physics [81] and electroweak precision observables [82], and the XENON100 and LUX searches for spin-independent dark-matter scattering [83, 84].

We find that the NUHM2, despite its freedom in the choices of $M_A$ and $\mu$, is unable to alleviate significantly the tension between the absence to date of SUSY at the LHC and the supersymmetric interpretation of $(g-2)_\mu$ that had been found previously in the CMSSM and NUHM1. We find that the minimum $\chi ^2/\mathrm{dof} = 32.5/21$ in the NUHM2, to be compared with $\chi ^2/\mathrm{dof} = 35.0/23$ in the CMSSM and $\chi ^2/\mathrm{dof} = 32.7/22$ in the NUHM1. A novel feature of the best NUHM2 fit is that the preferred regions of the NUHM2 parameter space have negative SUSY-breaking scalar masses squared for squarks and sleptons, $m_0^2 < 0$, as well as $m^2_{H_u} < m^2_{H_d} < 0$.

It is quite possible that SUSY-breaking scalar masses are negative at the GUT scale and yet, when run down to the weak scale, there are no tachyonic scalars in the theory. This extension to the CMSSM parameter space in the context of a gravitino LSP was considered in [85, 86], in mirage-mediated models in [87], and in recent post-Higgs gauge mediation constructions [88]. These models as well as NUHM2 models with $m^2_{H_u} < 0$ and $m^2_{H_d} < 0$ are potentially problematic due to the presence of charge- and colour-breaking minima, particularly along F- and D-flat directions [89, 90]. However, so long as the standard electroweak vacuum is long-lived, the relevance of other vacua becomes a cosmological question related to our position in field space after inflation. For a discussion of cosmological issues associated with such tachyonic soft SUSY-breaking mass parameters; see [91].

As an output of our analysis, we compare the one-dimensional likelihood functions for sparticle masses and other observables in the NUHM2 with those found previously in the CMSSM and NUHM1. The 95 % CL lower limits on the gluino, squark, stop and stau masses are not very different in the NUHM2 from those found previously in the CMSSM and NUHM1. However, the distinction found in those models between low- and high-mass regions of their respective parameter spaces is largely lost in the NUHM2 because of its greater flexibility in satisfying the dark-matter constraint. In addition to sparticle masses, we also present NUHM2 predictions for $\mathrm{BR}(B_s \rightarrow \mu ^+\mu ^-)$ and the spin-independent dark-matter scattering cross section, $\sigma ^\mathrm{SI}_p$.

## Analysis procedure

We follow closely the procedure described in [19]. Our treatment of the non-LHC constraints is identical with the treatment in [19], and we treat the top quark mass and the strong coupling as nuisance parameters with Gaussian priors: $m_t= 173.2 \pm 0.9 \,\, \mathrm {GeV}$ and $\alpha _s (M_Z) = 0.1185 \pm 0.0006$. We again use the MultiNest algorithm to sample the NUHM2 parameter space, just as we did previously for the CMSSM and NUHM1 models. As mentioned in the Introduction, we use a NUHM2 sample comprising ${\sim }4 \times 10^8$ points, with the aim of sampling adequately features of the six-dimensional NUHM2 parameter space $\{m_0, m_{1/2}, m_{H_u}, m_{H_d}, A_0, \tan \beta \}$, ensuring in particular that all high-likelihood regions are identified and well characterised. We sample the ranges $-1333 \,\, \mathrm {GeV}< m_0 < 4000 \,\, \mathrm {GeV}$, $0 < m_{1/2} < 4000 \,\, \mathrm {GeV}$, $- 5\times 10^7 \,\, \mathrm {GeV}^2 < m_{H_u}^2, m_{H_d}^2 < 5\times 10^7 \,\, \mathrm {GeV}^2$, $- 8000 \,\, \mathrm {GeV}< A_0 < 8000 \,\, \mathrm {GeV}$ and $2 < \tan \beta < 68$. (Here and subsequently, negative values of $m_0$ should be understood as $m_0 \equiv \mathrm{Sign}(m_0^2) \sqrt{|m_0^2|} < 0$, and we use analogous definitions for negative values of $m_{H_u}$ and $m_{H_d}$.) The parameter ranges are scanned by dividing the range of $m_0$ into 4 segments, and the ranges of $m_{1/2}, m_{H_u}$ and $m_{H_d}$ into 3 segments each, yielding a total of 108 boxes. Their boundaries are smeared using a Gaussian function so as to sample the NUHM2 parameter space smoothly, which also provides some information beyond the nominal sampling range, as we discuss later in the case of $m_{H_u}$ and $m_{H_d}$.

We merge this dedicated sample of the NUHM2 parameter space with the samples of the CMSSM and NUHM1 parameter spaces used in [19]. The latter are subspaces of the full NUHM2 parameter space, and the CMSSM and NUHM1 points provide supplementary sampling of the likelihood function of the NUHM2.

We construct a global likelihood function that receives contributions from the usual electroweak precision observables, as well as B-decay measurements such as BR($b \rightarrow s \gamma$), BR($B_u \rightarrow \tau \nu _\tau$) and $\mathrm{BR}(B_s \rightarrow \mu ^+\mu ^-)$. Bounds on their experimental values as well as those on the cosmological dark matter density, the cross section for spin-independent dark-matter scattering from the LUX experiment and the LHC search for supersymmetric signals are given in [77], with updates detailed in [92]. The observables we use, as well as the values and errors we assume, are given in Table 1, with references to their sources.

**Table 1:** List of experimental constraints used in this work, including experimental and (where applicable) theoretical errors: supersymmetric theory uncertainties in the interpretations of one-sided experimental limits are indicated by [$\ldots$]

Observable	Source Th./Ex.	Constraint
$m_t$ [GeV]	[93]	$173.2 \pm 0.87$
$\Delta \alpha _\mathrm{had}^{(5)}(M_Z)$	[93]	$0.02756 \pm 0.00010$
$M_Z$ [GeV]	[93, 94]	$91.1875\pm 0.0021$
$\Gamma _{Z}$ [GeV]	[93–96]	$2.4952\pm 0.0023\pm 0.001_\mathrm{SUSY}$
$\sigma _\mathrm{had}^{0}$ [nb]	[93–96]	$41.540\pm 0.037$
$R_l$	[93–96]	$20.767\pm 0.025$
$A_\mathrm{fb}(\ell )$	[93–96]	$0.01714\pm 0.00095$
$A_{\ell }(P_\tau )$	[93–96]	0.1465 $\pm$ 0.0032
$R_\mathrm{b}$	[93–96]	0.21629 $\pm$ 0.00066
$R_\mathrm{c}$	[93–96]	0.1721 $\pm$ 0.0030
$A_\mathrm{fb}({b})$	[93–96]	0.0992 $\pm$ 0.0016
$A_\mathrm{fb}({c})$	[93–96]	0.0707 $\pm$ 0.0035
$$ A_{b}$$	[93–96]	0.923 $$\pm $$ 0.020
$$ A_{c}$$	[93–96]	0.670 $$\pm $$ 0.027
$A_\ell (\mathrm{SLD})$	[93–96]	0.1513 $\pm$ 0.0021
$\sin ^2 \theta _\mathrm{w}^{\ell }(Q_\mathrm{fb})$	[93–96]	0.2324 $\pm$ 0.0012
$M_W$ [GeV]	[93–96]	$80.385 \,\pm \, 0.015\pm \,0.010_\mathrm{SUSY}$
$a_{\mu }^\mathrm{EXP} - a_{\mu }^\mathrm{SM}$	[65–74]	$(30.2 \pm 8.8 \pm 2.0_\mathrm{SUSY})\times 10^{-10}$
$M_h$ [GeV]	[97–101]/[93]	$125.7 \pm 0.4 \pm 1.5_\mathrm{SUSY}$
BR$_\mathrm{b \rightarrow s \gamma }^\mathrm{EXP/SM}$	[81, 102–106]	$1.089 \pm 0.070_\mathrm{EXP}$
		$$\pm \,\, 0.080_\mathrm{SM}\pm 0.050_\mathrm{SUSY}$$
BR$(B_{s,d} \rightarrow \mu ^{+} \mu ^{-})$	[14–16, 107–111]	CMS & LHCb
BR$_\mathrm{B \rightarrow \tau \nu }^\mathrm{EXP/SM}$	[81, 112–114]	$1.39 \pm 0.28_\mathrm{EXP} \pm 0.13_\mathrm{SM}$
$\mathrm{BR}_{B \rightarrow X_s \ell \ell }^\mathrm{EXP/SM}$	[81, 115]	$0.99 \pm 0.32$
BR$_{K \rightarrow \mu \nu }^\mathrm{EXP/SM}$	[112, 113, 116]	$1.008 \pm 0.014_\mathrm{EXP+TH}$
BR$_{K \rightarrow \pi \nu \bar{\nu }}^\mathrm{EXP/SM}$	[117, 118]	${<} 4.5$
$\Delta M_{B_s}^\mathrm{EXP/SM}$	[117, 119]	$0.97 \pm 0.20_\mathrm{SM}$
$\frac{\Delta M_{B_s}^\mathrm{EXP/SM}}{\Delta M_{B_d}^\mathrm{EXP/SM}}$	[112, 113, 119]	$0.86 \pm 0.14_\mathrm{SM}$
$\Delta \epsilon _K^\mathrm{EXP/SM}$	[81, 117]	$1.14 \pm 0.10_\mathrm{EXP+TH}$
$\Omega _\mathrm{CDM} h^2$	[120–123]	$0.1186 \pm 0.0022 \pm 0.0120_\mathrm{SUSY}$
$\sigma ^\mathrm{SI}_p$	[84]	$(m_{\tilde{\chi }^0_{1}}, \sigma ^\mathrm{SI}_p)$ plane
jets + ${/\!\!E}_T$	[10]	$(m_0, m_{1/2})$ plane
$H/A, H^\pm$	[124, 125]	$(M_A, \tan \beta )$ plane

The contributions of these observables to the likelihood function are calculated within the MasterCode framework [126]. This incorporates a code for the electroweak observables based on [95, 96] as well as the SoftSUSY [127], FeynHiggs [97–101, 128], SuFla [112, 113], SuperIso [129–131], MicrOMEGAs [120–122] and SSARD [132] codes, using the SUSY Les Houches Accord [133, 134]. The ATLAS and CMS measurements of the Higgs mass, $M_h$, are interpreted using FeynHiggs 2.10.0 [128] to calculate $M_h$ and, as in [19] we allow conservatively for a theoretical uncertainty of 1.5 GeV^3^ at each point in the NUHM2 parameter space.^4^ The improvements recently incorporated into FeynHiggs [97–101] yield an upward shift of $M_h$ for scalar top masses in the (multi-)TeV range and reduce the theoretical uncertainty in the Higgs mass calculation [139], which is nevertheless significantly larger than the variations in the best-fit Higgs mass since its discovery and the differences between the values reported by ATLAS and CMS.

We incorporate here the public results of the search for jets + ${/\!\!E}_T$ events without leptons using the full ATLAS Run 1 data set of ${\sim }20$/fb at 8 TeV [10], which has greater sensitivity to the relevant parts of the NUHM2 parameter space than searches including leptons and/or $b$ quarks.^5^ Experimental searches for jets + ${/\!\!E}_T$ events are typically analysed within the framework of the CMSSM for some fixed $A_0$ and $\tan \beta$. The applicability of these analyses to other $A_0$ and $\tan \beta$ values, as well as to constraining the NUHM1,2, requires some study and justification. One issue is that, for any specific set of values of $m_0$, $m_{1/2}$, $A_0$ and $\tan \beta$, the sensitivities of ATLAS and CMS to jets + ${/\!\!E}_T$ events might depend on the degree of non-universality in the NUHM1,2. A second issue is that the range of $m_0$ in the NUHM2 that is consistent with the $\tilde{\chi }^0_{1}$ LSP requirement depends on the degrees of non-universality. Specifically, this requirement is compatible with $m^2_0 < 0$ in the NUHM2, a possibility that is absent for the CMSSM, but can occur in the NUHM1 for $m_{1/2} \gtrsim 2000\,\, \mathrm {GeV}$ when $m^2_{H_d} = m^2_{H_u} < 0$ and dominates over $m_0^2$ in the renormalisation-group evolution. In the NUHM2 it is even easier to obtain $m^2_0 < 0$ and remain compatible with a neutralino LSP, because a combination of soft supersymmetry-breaking parameters known as $S$ (defined below) may be non-zero.

Since the ATLAS experiment quotes limits only for the CMSSM with $m^2_0 > 0$, we rely on a previous dedicated study of the jets + ${/\!\!E}_T$ search at 7 TeV [92], made using the Delphes [140] generic simulation package with a ‘card’ to emulate the performance of the ATLAS detector, that showed that the LHC results could be extrapolated to $m_0^2 < 0$. As shown in Figs. 2, 3 and 4 of [92], his study confirmed that ${/\!\!E}_T$ constraints in the $(m_0, m_{1/2})$ plane of the CMSSM are relatively insensitive to $\tan \beta$ and $A_0$, as stated in [141], and that the ${/\!\!E}_T$ constraints are also quite insensitive to the degrees of non-universality in the NUHM1,2, with any variations in the sensitivity being smaller than the uncertainties in our simulation. Specifically, it was found that the 95 % CL bounds in the $(m_0, m_{1/2})$ plane of the CMSSM were approximately independent of $A_0$ and $\tan \beta$, as also stated by CMS [142]; the same was true for $m_{H_u}^2 = m_{H_d}^2 \ne m_0^2$ in the NUHM1, and also for $m_{H_u}^2 \ne m_{H_d}^2 \ne m_0^2$ in the NUHM2. The same is expected to be true for the 8-TeV ATLAS jets + ${/\!\!E}_T$ search [10] used here, which uses a similar event selection to the ATLAS 7-TeV data studied in [92].

Finally, we also incorporate here the most recent constraints on $A/H$ production from ATLAS and CMS [124, 125], using the same approach as in [19].

## Analysis of the NUHM2 parameter space

### Scalar-mass parameters and renormalisation

Before discussing our results for the NUHM2, we briefly review another important difference between this model and its more constrained relatives. When $m_{H_u}^2 \ne m_{H_d}^2$, the quantity [143]

1 $$\begin{aligned} S&\equiv \frac{g_1^2}{4} \big ( m_{H_u}^2 \!\!- m_{H_d}^2 \!+\! 2 \big ( m_{\widetilde{Q}_L}^2 \!\!- m_{\widetilde{L}_L}^2\!\!- 2 m_{\widetilde{u}_R}^2 \!+ m_{\widetilde{d}_R}^2 \!+ m_{\widetilde{e}_R}^2 \big )\nonumber \\&+ \, \big ( m_{\widetilde{Q}_{3L}}^2 - m_{\widetilde{L}_{3L}}^2 - 2 m_{\widetilde{t}_R}^2 + m_{\widetilde{b}_R}^2 + m_{\widetilde{\tau }_R}^2 \big )\big ) \end{aligned}$$

is non-zero. In both the CMSSM and NUHM1, $S=0$ and is a fixed point of the RGEs at the one-loop level and remains zero at any scale [144, 145]. However, in the NUHM2, with $m^2_{H_u} \ne m^2_{H_d}$, $S \ne 0$ at the GUT scale, as seen in (1), which can cause the low-energy spectrum to differ significantly from that in the CMSSM or NUHM1. For example, consider the renormalisation-group equation for the $\tau _R$ mass:

2 $$\begin{aligned} \frac{\mathrm{d} m_{\widetilde{\tau }_R}^2}{\mathrm{d}t}&= \frac{1}{8 \pi ^2} (-4 g_1^2 M_1^2 \nonumber \\&+\,\,2 h_\tau ^2 ( m_{\widetilde{L}_{3L}}^2 + m_{\widetilde{\tau }_R}^2 +m_1^2 + A_\tau ^2)+4S). \end{aligned}$$

When $S<0$, the evolution of $m^2_{\tau _R}$ receives a positive contribution as it runs down from the GUT scale to the electroweak scale. As a result, ensuring a neutralino LSP becomes a generic possibility even when $m^2_0 < 0.$ 6 Furthermore, the masses of left-handed sleptons may run to lighter values than their right-handed counterparts, allowing for new coannihilation channels to regulate the neutralino relic density [78, 79], or larger contributions to $(g-2)_\mu$.

### Model parameter planes

*The* $$(m_0, m_{1/2})$$ *plane:*

**Fig. 1:**
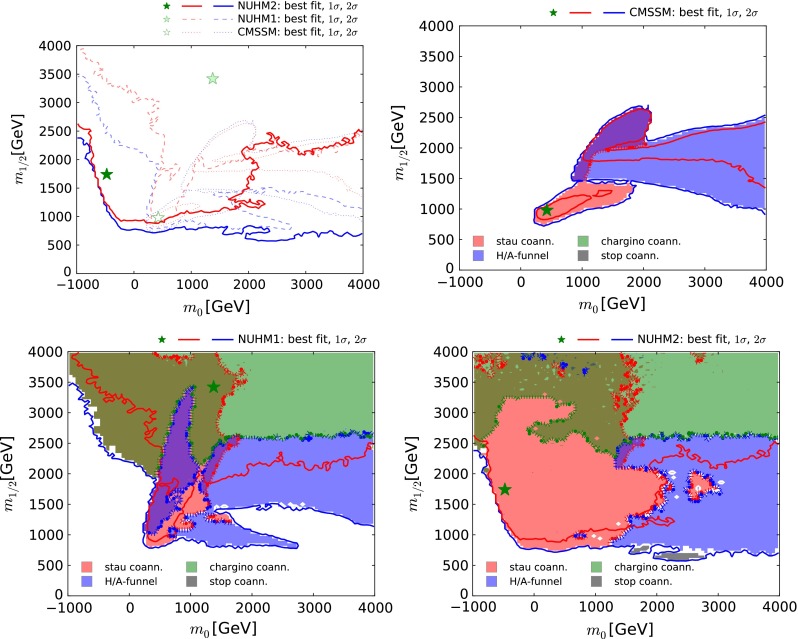
*Upper left* the $(m_0, m_{1/2})$ planes in the NUHM2, CMSSM and NUHM1. The results of the fit in the NUHM2 are indicated by *solid lines* and *filled green stars*, and those of our previous fits to the CMSSM and NUHM1 by *dotted* and *dashed lines* as well as open and *shaded green stars*, respectively. In all cases, the *red lines* denote $\Delta \chi ^2 = 2.30$ (${\sim }68$ % CL) contours, and the *blue lines* denote $\Delta \chi ^2 = 5.99$ (${\sim }95$ % CL) contours. *Upper right* the dominant mechanisms (3) fixing the dark-matter density $\Omega _\chi h^2$ in the CMSSM. *Lower left* the same for the NUHM1. *Lower right* the same for the NUHM2. Stau coannihilation regions are *shaded pink*, rapid $A/H$ annihilation funnel regions are *shaded blue*, $\tilde{\chi }^\pm _{1}$ coannihilation regions are *shaded green*, stop coannihilation regions are *shaded grey*. Regions where more than one of these conditions are satisfied are *shaded in darker colours*

We first present results for the $(m_0, m_{1/2})$ plane shown in Fig. 1. We denote the best-fit point by a filled green star and the $\Delta \chi ^2 = 2.30$ and 5.99 contours by solid red and blue contours, respectively. These would correspond to 68 and 95 % CL contours if the errors were Gaussian. In the upper left panel of Fig. 1 we also show the best-fit points in the NUHM1 and CMSSM (shaded and open green stars), and the 68 and 95 % CL contours in these models (dashed and dotted red and blue contours, respectively). It is apparent from Fig. 1 that the extents of these contours that the $\chi ^2$ function for the NUHM2 is quite shallow, and we emphasise that the best-fit point and other details of the $\chi ^2$ function should not be over-interpreted.

We see that the 68 % CL NUHM2 region in the upper left panel of Fig. 1 extends in a lobe down to $m_{1/2} \sim 300$ to $2000 \,\, \mathrm {GeV}$ for $-500 \,\, \mathrm {GeV}\lesssim m_0 \lesssim 2000 \,\, \mathrm {GeV}$, whereas $m_0$ is relatively unrestricted for $m_{1/2} \gtrsim 2500 \,\, \mathrm {GeV}$. At the 95 % CL we find $m_{1/2} \gtrsim 500 \,\, \mathrm {GeV}$ for $m_0 \gtrsim 0$. The best-fit point in the NUHM2 has $m_0 \sim - 500 \,\, \mathrm {GeV}$ and $m_{1/2} \sim 1800 \,\, \mathrm {GeV}$. The LHC ${/\!\!E}_T$ search with the most impact on the parameter space is that with jets and zero leptons, which constrains the NUHM2 parameter space most when $m_0 \lesssim 1500 \,\, \mathrm {GeV}$. As already mentioned, we have verified previously [92] that this constraint is approximately independent of the other NUHM2 parameters in the $(m_0, m_{1/2})$ region of interest. Searches for events with $b$-jets and/or leptons have greater sensitivity when $m_0 \gtrsim 1500 \,\, \mathrm {GeV}$, but are important only outside the 95 % CL contour, at lower $m_{1/2}$, so we have not studied in detail their sensitivity to the model parameters.

In the case of the NUHM1, the range of $m_0$ where low values of $m_{1/2} \lesssim 2000 \,\, \mathrm {GeV}$ are allowed at the 68 % CL (within the dashed red contour in Fig. 1) is much smaller, being limited to $200 \,\, \mathrm {GeV}\lesssim m_0 \lesssim 1000 \,\, \mathrm {GeV}$. The case of the CMSSM is much more restrictive, with only a small part of the 68 % CL region (within the dotted red contour in Fig. 1) with $300 \,\, \mathrm {GeV}\lesssim m_0 \lesssim 1300 \,\, \mathrm {GeV}$ appearing when $m_{1/2} \lesssim 1800 \,\, \mathrm {GeV}$. Moreover, this case has a largest allowed value of $m_{1/2} \sim 2500 \,\, \mathrm {GeV}$ at the 95 % CL, whereas we observe no upper bound on $m_{1/2}$ in either the NUHM1 or the NUHM2.

*The dark-matter constraint:*

The dark-matter density constraint is less restrictive in the NUHM2 than in the NUHM1 and, particularly, the CMSSM. In the regions of interest, the dark-matter density is generally brought down into the range allowed by cosmology through enhancement of (co-)annihilation processes due to particular properties of the spectrum. In the other panels of Fig. 1 we use different colours of shading to visualise the impacts of these processes, by displaying areas of the 95 % CL regions in the $(m_0, m_{1/2})$ planes where the following conditions are satisfied:

3 $$\begin{aligned}&{\tilde{\tau }_1}\hbox { coannihilation (pink):}\quad \frac{m_{\tilde{\tau }_1}}{m_{\tilde{\chi }^0_{1}}} - 1<0.15,\nonumber \\&A/H \hbox { funnel (blue):}\quad \left| \frac{M_A}{2m_{\tilde{\chi }^0_{1}}} - 1 \right| <0.2, \nonumber \\&\tilde{\chi }^\pm _{1} \hbox { coannihilation (green):}\quad \frac{m_{\tilde{\chi }^\pm _{1}}}{m_{\tilde{\chi }^0_{1}}} - 1<0.1,\nonumber \\&{\tilde{t}_1} \hbox { coannihilation (grey):}\quad \frac{m_{\tilde{t}_1}}{m_{\tilde{\chi }^0_{1}}} - 1<0.2. \end{aligned}$$

each of which is surrounded by a dotted contour. Regions where more than one of these conditions are satisfied are shaded in darker colours. We have also explored the focus-point [146–148] criterion $| \mu /m_{\tilde{\chi }^0_{1}} - 1| < 0.3$, and found that it is not relevant in the displayed portions of the $(m_0, m_{1/2})$ planes. We note that the criteria above are approximate, being intended only to serve as guides to the different regions in the $(m_0,m_{1/2})$ planes.

We see in the upper right panel of Fig. 1 that the low-mass region of the CMSSM is in the stau coannihilation region [149–157] (pink shading) and its high-$m_0$ region (blue shading) is in the funnel region where the LSPs annihilate rapidly through the s-channel heavy Higgs resonances $A/H$ [43–47]. The best-fit CMSSM point now lies in the stau coannihilation region: the difference from the low-mass best-fit point found in [19] is due to using the updated ATLAS jets + ${/\!\!E}_T$ constraint [10]. The current CMSSM best-fit point is very similar to the previous local best fit in the low-mass region. We also see for $1000 \,\, \mathrm {GeV}\lesssim m_0 \lesssim 2000 \,\, \mathrm {GeV}$ and $m_{1/2} \gtrsim 2000 \,\, \mathrm {GeV}$ (shaded purple) a CMSSM region where both the stau-coannihilation and funnel criteria are satisfied.

In the NUHM1, as seen in the lower left panel of Fig. 1 it is possible to satisfy the $\Omega _\chi h^2$ constraint for larger values of $m_{1/2}$ than are possible in the CMSSM, thanks to the extra degree of freedom associated with the soft SUSY-breaking contribution to the Higgs masses. In the low-mass NUHM1 region, the relic density is again determined by stau coannihilation (pink shading), whereas at large $m_0$ and $m_{1/2} \lesssim 2500 \,\, \mathrm {GeV}$ the rapid annihilation via the $A/H$ funnel (blue shading) is important. The NUHM1 best-fit point is in a high-mass region where $\Omega _\chi h^2$ is determined by coannihilations of nearly degenerate $\tilde{\chi }^0_{1}$, $\tilde{\chi }^\pm _{1}$ and $\tilde{\chi }^0_{2}$ [157–161] (green shading), since $\mu \ll m_{1/2}$ and the LSP is nearly a pure higgsino. There is also a region where both stau and $\tilde{\chi }^\pm _{1}$ coannihilations are important (dark green shading).

In the case of the NUHM2, all four of the mechanisms (3) come into play, as we see in the lower right panel of Fig. 1. As in the cases of the CMSSM and NUHM1, there are regions where stau coannihilation (pink), rapid annihilation via $A/H$ bosons (blue) and $\tilde{\chi }^\pm _{1}$ coannihilations (green) are important, as well as a region where both stau and $\tilde{\chi }^\pm _{1}$ coannihilations are important (dark green). We also see two small bands with $(m_0, m_{1/2}) \sim (2000, 600) \,\, \mathrm {GeV}$ where stop coannihilation [162–167] is important.

**Fig. 2:**
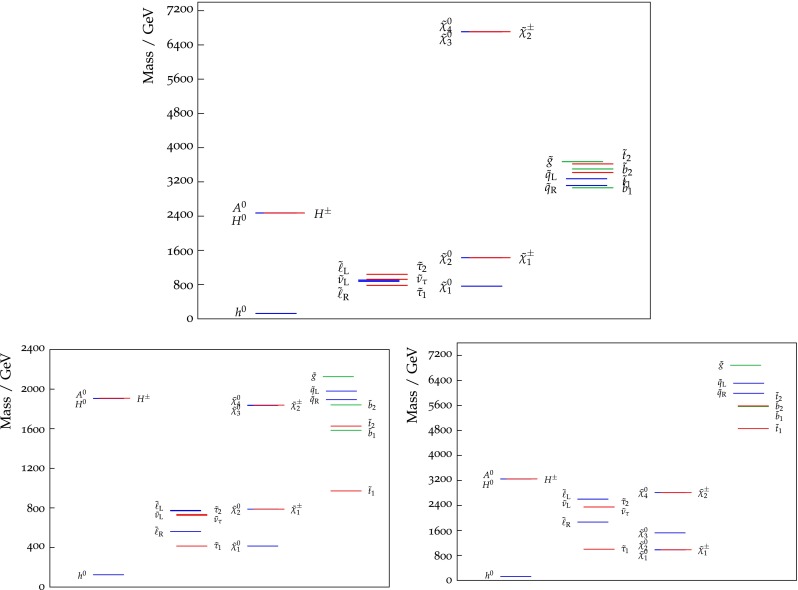
The spectrum at the best-fit point found in our frequentist fit to the NUHM2 (*top*), and to the CMSSM (*bottom left*) and the NUHM1 (*bottom right*)

Our best-fit point for the NUHM2 has $m^2_0 < 0$ in the pink region where the relic density is fixed by stau coannihilation. As can be seen in Fig. 2, the LSP and the lighter stau are indeed very nearly degenerate at this point, with the other sleptons only slightly heavier but the other sparticles significantly more massive. Also, $M_A\gg 2 m_{\tilde{\chi }^0_{1}}$, so there is no significant enhancement of LSP annihilations via direct-channel resonances. We emphasise, however, that the NUHM2 spectrum is poorly determined, and that this and other processes play important roles in other parts of the NUHM2 parameter space. We find $M_h= 124.8~\,\, \mathrm {GeV}$ at the best-fit point. For comparison, the lower panels of Fig. 2 display the best-fit spectra in the CMSSM (left) and the NUHM1 (right). In the case of the CMSSM, the best-fit point is also in the stau coannihilation region, whereas the best NUHM1 fit is in a region where both stau and $\tilde{\chi }^\pm _{1}$ coannihilations are important.

*Other parameter planes:*

Figure 3 displays the $(m_0, \tan \beta )$ plane (left) and the $(\tan \beta , m_{1/2})$ plane (right) in the NUHM2, CMSSM and NUHM1. In both panels, we see that a large range $5 \lesssim \tan \beta \lesssim 60$ is allowed at the 68 % CL (solid red contour).^7^ The range of $\tan \beta$ within the 68 % CL region is restricted to values ${\lesssim } 40$ for the lower-mass lobe in Fig. 1 where $m_0 \lesssim 1000 \,\, \mathrm {GeV}$ and $m_{1/2} \lesssim 2500 \,\, \mathrm {GeV}$. Once again, we see that the additional freedom of being able effectively to choose $\mu$ and $M_A$ independently allows solutions with the correct relic density over a wider range of the parameters $m_0, m_{1/2}$ and $\tan \beta$. The region of the $(m_0, \tan \beta )$ plane with $|m_0| \lesssim 1000 \,\, \mathrm {GeV}$ is generally in the stau coannihilation region, whereas in the region at larger $m_0$ and $\tan \beta \lesssim 40$ $\tilde{\chi }^\pm _{1}$ coannihilation is important. The prominent horizontal lobe in the left-hand plot at $\tan \beta \sim 50$ is associated with the $A$-funnel region.

**Fig. 5:**
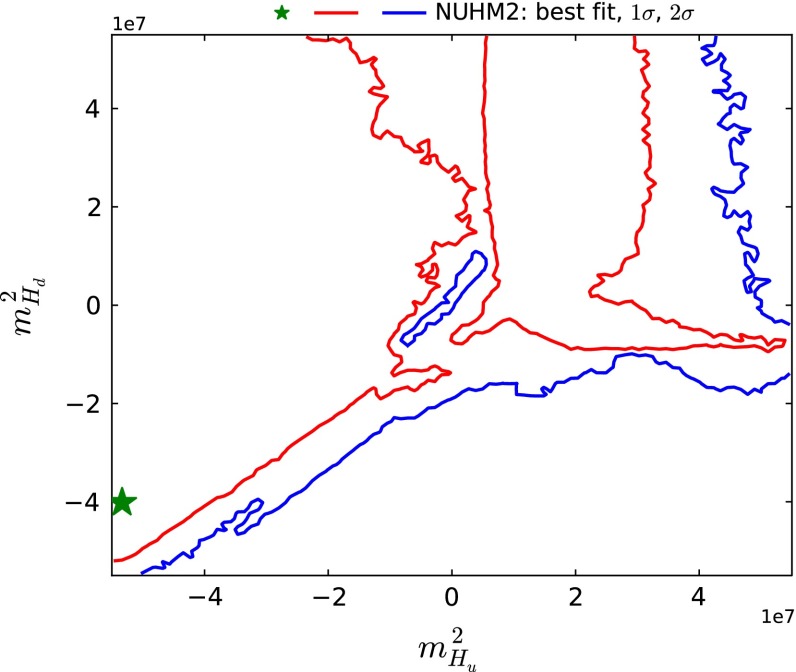
The $(m_{H_u}^2, m_{H_d}^2)$ plane in the NUHM2. The *star* and *contours* have the same significations as in Fig. 1

Figure 4 displays the $(m_0, m_{H_u}^2)$ and $(m_0, m_{H_d}^2)$ planes of the NUHM2 (left and right panels, respectively). We see again that the best-fit point has $m_0 < 0$, and that both $m_{H_{u,d}}^2 < 0$ are favoured, with a preference for $m_{H_u}^2 < m_{H_d}^2.$ 8 The reason for this preference can be understood from (1). To obtain a neutralino LSP, we require $S < 0$, which then requires $m_{H_u}^2 < m_{H_d}^2$. In general, stau coannihilation is most important when $m_{H_u}^2$ or $m_{H_d}^2 \lesssim 0$, whereas $\tilde{\chi }^\pm _{1}$ coannihilation is more important when $m_{H_u}^2$ or $m_{H_d}^2 \gtrsim 0$. Figure 5 displays the $(m_{H_u}^2, m_{H_d}^2)$ plane for the NUHM2, where we see that the best-fit point has $m_{H_u}^2 < m_{H_d}^2 < 0$. However, we emphasise that the global likelihood function is quite flat in $m_{H_{u,d}}^2$, and the most reliable statement that can be made is that the quadrant $m_{H_u}^2 > 0, m_{H_d}^2 < 0$ is the least favoured. When $m_{H_u}^2 \lesssim 0$, stau coannihilation is important for $m_{H_d}^2 \gtrsim m_{H_u}^2$, but the $A/H$ funnel is important when $m_{H_d}^2 \sim m_{H_u}^2$. When $m_{H_u}^2 \gtrsim 0$, $\tilde{\chi }^\pm _{1}$ coannihilation is important for $m_{H_d}^2 \gtrsim 0$ whereas stop coannihilation becomes important for $m_{H_d}^2 < 0$.

**Fig. 6:**
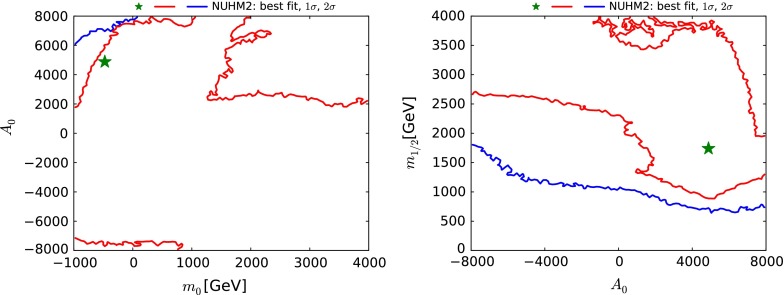
The $(m_{0}, A_0)$ plane (*left panel*) and the $(m_{1/2}, A_0)$ plane (*right panel*) in the NUHM2. The significations of the *solid lines* and *filled stars* are the same as in Fig. 1

Figure 6 displays the $(m_{0}, A_0)$ plane (left) and the $(A_0, m_{1/2})$ plane (right) for the NUHM2. The fit does not exhibit any overall preference for a sign of $A_0$. However, we see that negative values of $A_0$ are generally preferred when $m_0$ and $m_{1/2}$ are large, whereas the low-mass lobe in Fig. 1 is generally associated with positive values of $A_0.$ 9 This tendency is driven by the value of $M_h$ measured at the LHC.

**Fig. 7:**
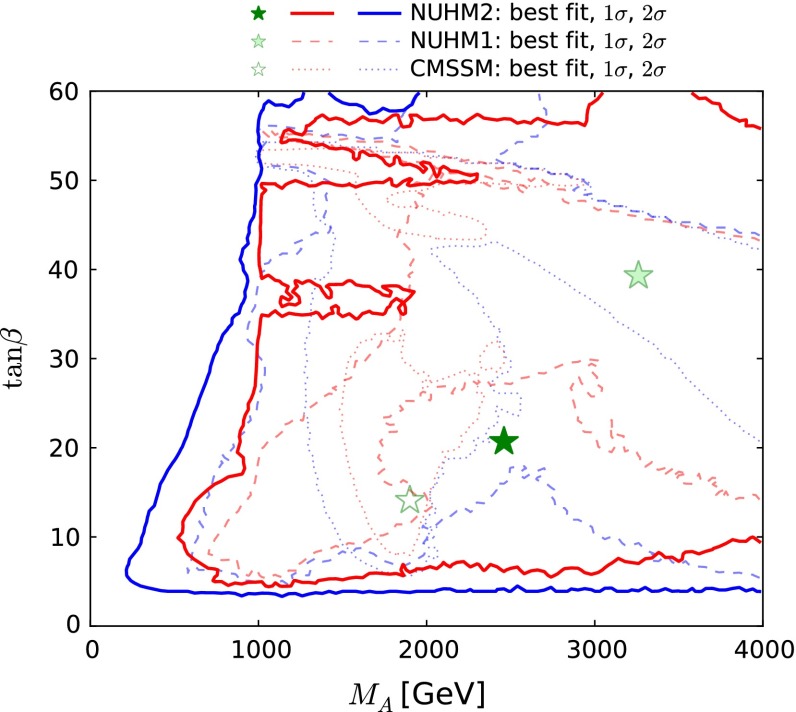
The $(M_A, \tan \beta )$ plane in the NUHM2, CMSSM and NUHM1. The *lines* and *stars* have the same significations as in Fig. 1

Figure 7 displays the $(M_A, \tan \beta )$ plane in the NUHM2 (solid lines), CMSSM (dashed lines) and NUHM1 (dotted lines). In the NUHM2 we see a 95 % CL lower limit on $M_A$ that increases from ${\sim } 200 \,\, \mathrm {GeV}$ when $\tan \beta \sim 5$ to $1000 \,\, \mathrm {GeV}$ when $\tan \beta \sim 50$, which is essentially determined by the $H/A \rightarrow \tau \tau$ constraint [124, 125], with cut-outs due to the $\chi ^2$ penalties as different mechanisms for satisfying the $\Omega _\chi h^2$ constraint come into play or become ineffective. The best-fit value of $M_A\sim 2500 \,\, \mathrm {GeV}$, but the global $\chi ^2$ function is very flat, and this model parameter is not well determined, and could be as low as 500 GeV at the 68 % CL. We find a 95 % CL lower limit $\tan \beta \gtrsim 4$, which is quite insensitive to the value of $M_A$. We find that $\tilde{\chi }^\pm _{1}$ coannihilation is generally important for $M_A\lesssim 2000 \,\, \mathrm {GeV}$, whereas stau coannihilation is important for $M_A\gtrsim 2000 \,\, \mathrm {GeV}$. The $A/H$ funnel becomes important for $M_A\sim 2000 \,\, \mathrm {GeV}$, and also for $\tan \beta \gtrsim 50$.

### Summary of NUHM2 global fit

Table 2 summarises our results for the our best-fit points in a global fit to the NUHM2, compared with fits in the NUHM1 and the CMSSM using the same post-LHC Run 1 data set. We see that the total $\chi ^2$ in the best NUHM2 fit is lowered by only $\Delta \chi ^2=0.2$ from the best NUHM1 fit, so the extra parameter in the NUHM2 does not provide a significant advantage. According to the F-test, there is a 77 % chance that the data are represented better by the NUHM1 than by the CMSSM, whereas there is only a 28 % chance that the NUHM2 is an improvement on the NUHM1, and a 78 % chance that the NUHM2 represents the data better than the CMSSM. None of these can be regarded as significant.

**Table 2:** The best-fit points found in global fits in the CMSSM, the NUHM1 and the NUHM2, using the same experimental constraints (and their theoretical interpretations): the difference in the CMSSM best-fit from that found in [19] is due to using the updated ATLAS jets + ${/\!\!E}_T$ constraint [10]. We note that the overall likelihood functions in all the models are quite flat, so that the precise locations of the best-fit points are not very significant, and for this reason we do not quote uncertainties. The $p$-values quoted would have the interpretations of probabilities if the likelihood functions given by the $\chi ^2$ statistics were Gaussian

Model	$\chi ^2$/dof	$p$-value (%)	$m_0$ (GeV)	$m_{1/2}$ (GeV)	$A_0$ (GeV)	$\tan \beta$	$m_{H_u}^2$ (GeV$^2$)	$m_{H_d}^2$ (GeV$^2$)
CMSSM	35.0/23	5.2	420	970	3000	14	${=} m_0^2$	${=} m_0^2$
NUHM1	32.7/22	6.6	1380	3420	$-$3140	39	$1.33 \times 10^7$	${= }m_{H_u}^2$
NUHM2	32.5/21	5.2	$-$490	1730	4930	21	$- 5.28 \times 10^7$	$- 4.03 \times 10^7$

We note that the NUHM2 best-fit value of $m_0$ is small and negative, and that it is accompanied by values of $m_{H_u}^2$ and $m_{H_d}^2$ that are also negative and larger in magnitude.^10^ We have checked the vacuum stability of the best-fit point using the Vevacious code [168], finding that it is metastable. The best-fit value of $m_{1/2}$ in the NUHM2 lies significantly beyond the direct lower limit from sparticle searches at the LHC. We also find that a positive value of $A_0$ is preferred, in contrast to the NUHM1 and the CMSSM which have much larger values of $m_0$ and $m_{1/2}$ at their best fit points. That said, we repeat that the likelihood functions are extremely shallow, and the 68 % ranges very large, so the best fit point should not be over-interpreted.

## Predictions for physical observables

We now turn to the predictions for physical observables that emerge from our frequentist analysis of the NUHM2 parameter space, and compare them with corresponding predictions from our previous analyses of the CMSSM and NUHM1 parameter spaces [19]. Since the CMSSM is a subset of the NUHM1, which is itself a subset of the NUHM2, $\chi ^2|_\mathrm{CMSSM} \ge \chi ^2|_\mathrm{NUHM1} \ge \chi ^2|_\mathrm{NUHM2}$ everywhere. However, this is not immediately visible in the plots below, in which we plot the difference $\Delta \chi ^2$ from the minimum value of $\chi ^2$ in that model shown in the Table. In general, after falling from high values at low masses, the $\Delta \chi ^2$ are generally flat at high masses. However, there are some features associated with, for example, transitions between different mechanisms for bringing the relic density into the allowed range, which we comment on in the discussion below.

### Sparticle masses

**Fig. 8:**
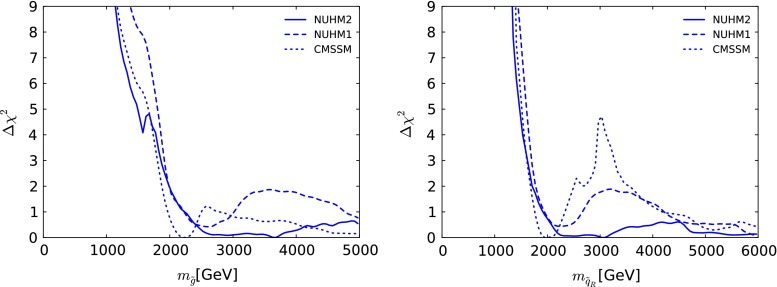
The $\Delta \chi ^2$ likelihood function in the NUHM2 (*solid line*) as a function of $m_{\tilde{g}}$ (*left panel*) and $m_{\tilde{q}_R}$ (*right panel*). The *dotted* (*dashed*) *lines* are for the corresponding fits in the CMSSM and NUHM1, respectively

In the left panel of Fig. 8 we display the $\Delta \chi ^2$ function in the NUHM2 (solid line) as a function of $m_{\tilde{g}}$. We see that $m_{\tilde{g}}\gtrsim 1.5 \,\, \mathrm {TeV}$ is preferred at the 95 % CL,^11^ as was the case in the CMSSM and NUHM1, and that the $\Delta \chi ^2$ function is quite flat for $m_{\tilde{g}}\gtrsim 2.5 \,\, \mathrm {TeV}$. The lower limit on $m_{\tilde{g}}$ is mainly due to the ATLAS jets + ${/\!\!E}_T$ constraint, counteracted to some extent by $(g-2)_\mu$: the LHC $M_h$ measurement plays no role. The best-fit point has $m_{\tilde{g}}\sim 3670~\,\, \mathrm {GeV}$ as seen also in Fig. 2. At low masses, the $\Delta \chi ^2$ function is similar to that for the CMSSM (dotted line), and also to the NUHM1(dashed line) when $m_{\tilde{g}}\lesssim 2 \,\, \mathrm {TeV}$. Above this mass, the difference between the $\Delta \chi ^2$ functions for the NUHM2 and the NUHM1 is largest for $3 \,\, \mathrm {TeV}\lesssim m_{\tilde{g}}\lesssim 5 \,\, \mathrm {TeV}$, where the extra freedom permitted when $m_{H_u}^2 \ne m_{H_d}^2$ allows the $\Omega _\chi h^2$ constraint to be satisfied with lower $\chi ^2$ penalties for the other observables.

The right panel of Fig. 8 displays the $\Delta \chi ^2$ likelihood as a function of $m_{\tilde{q}_R}$, defined here to be the average of the spartners of the right-handed components of the four lightest quarks. We see that $m_{\tilde{q}}\gtrsim 1.5 \,\, \mathrm {TeV}$ at the 95 % CL in the NUHM2, driven essentially by the ATLAS jets + ${/\!\!E}_T$ constraint, with a best-fit value $m_{\tilde{q}_R}\sim 3080~\,\, \mathrm {GeV}$ as seen also in Fig. 2, and that the $\Delta \chi ^2$ function in this model is very similar to those in the NUHM1 and CMSSM for $m_{\tilde{q}_R}\lesssim 2 \,\, \mathrm {TeV}$. However, the $\Delta \chi ^2$ functions in these models differ quite significantly for $2 \,\, \mathrm {TeV}\lesssim m_{\tilde{q}_R}\lesssim 4.5 \,\, \mathrm {TeV}$, reflecting the fact visible in Fig. 1 that the separation between the low- and high-mass regions becomes less pronounced as the Higgs mass universality is progressively relaxed. This can be traced back to the broader range of options for bringing the cold dark-matter density into the range preferred by cosmology.

**Fig. 9:**
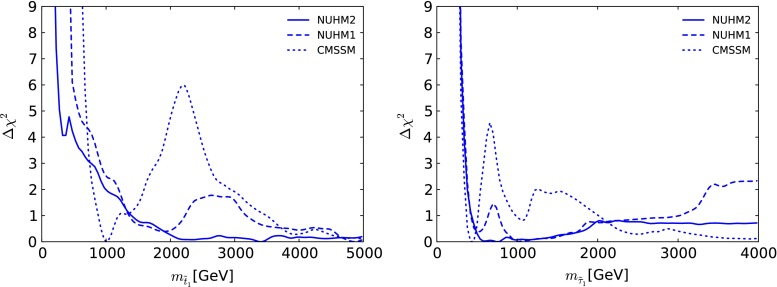
As in Fig. 8, for $m_{\tilde{t}_1}$ (*left panel*) and for $m_{\tilde{\tau }_1}$ (*right panel*)

In the left panel of Fig. 9 we display the $\Delta \chi ^2$ likelihood as a function of $m_{\tilde{t}_1}$. In this case the lower-mass limit is not driven by the ATLAS jets + ${/\!\!E}_T$ search. On the other hand, the $\Delta \chi ^2$ functions for these models are quite different at both larger and smaller $m_{\tilde{t}_1}$: lower masses are not so strongly disfavoured in the NUHM2, and the features found in the CMSSM at $m_{\tilde{t}_1} \sim 1 \,\, \mathrm {TeV}$ and ${\in }(2, 3) \,\, \mathrm {TeV}$ are not found in the NUHM2, whose $\Delta \chi ^2$ function falls almost monotonically as $m_{\tilde{t}_1}$ increases. This reflects again the fact that the low- and high-mass regions are less distinct in the NUHM2, whereas in the CMSSM the stau coannihilation region is quite separated from the $H/A$ funnel region at high masses. There are also some stop coannihilation points at low $m_{\tilde{t}_1}$. The best-fit point has $m_{\tilde{t}_1} \sim 3420~\,\, \mathrm {GeV}$ as seen also in Fig. 2. The right panel of Fig. 9 displays the $\Delta \chi ^2$ functions in the NUHM2, NUHM1 and CMSSM as functions of $m_{\tilde{\tau }_1}$. At low mass, we see that the $\Delta \chi ^2$ functions are almost identical in the three models, giving a lower bound $m_{\tilde{\tau }_1} \gtrsim 300 \,\, \mathrm {GeV}$ at the 95 % CL, driven by the ATLAS jets + ${/\!\!E}_T$ search. At intermediate masses, the $\chi ^2$ functions in the NUHM1 and NUHM2 are reduced by the operation of extra dark matter density reduction mechanisms, which are operative in the NUHM2 also at higher masses, but not in the NUHM1 The structures seen in the $\Delta \chi ^2$ functions for the NUHM1 (dashed line) and CMSSM (dotted line) are absent for the NUHM2, whose $\Delta \chi ^2$ function (solid line) has a shallow minimum at $m_{\tilde{\tau }_1} \sim 780 \,\, \mathrm {GeV}$.

**Fig. 10:**
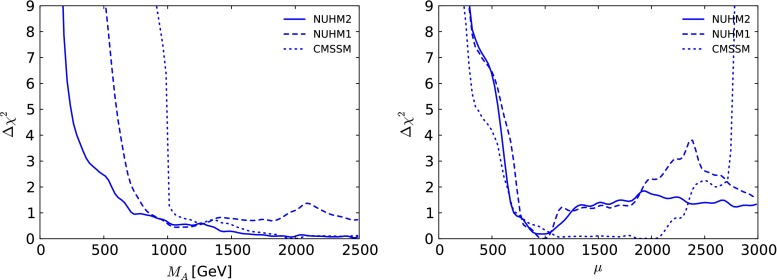
As in Fig. 8, for $M_A$ (*left panel*) and for $\mu$ (*right panel*)

The left panel of Fig. 10 displays the dependences of the $\Delta \chi ^2$ functions in the NUHM2, NUHM1 and CMSSM on $M_A$. We see that the $\Delta \chi ^2$ function for the NUHM2 is quite flat above ${\sim } 500 \,\, \mathrm {GeV}$, following a steep rise at lower masses and a 95 % CL lower limit $M_A\gtrsim 200 \,\, \mathrm {GeV}$, which is largely due to the $H/A \rightarrow \tau \tau$ constraint [124, 125] as mentioned previously. The best-fit point has $M_A\sim 2470~\,\, \mathrm {GeV}$ as seen also in Fig. 2. The right panel of Fig. 10 displays the corresponding $\Delta \chi ^2$ function for $\mu$. Like $M_A$, this extra degree of freedom in the NUHM2 is poorly constrained by current data.

**Fig. 11:**
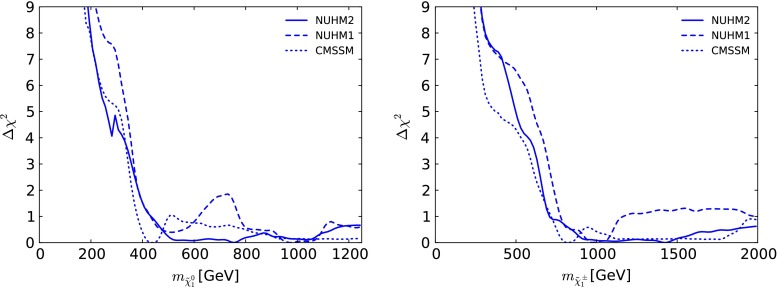
As in Fig. 8, for $m_{\tilde{\chi }^0_{1}}$ (*left panel*) and for $m_{\tilde{\chi }^\pm _{1}}$ (*right panel*)

Figure 11 displays the $\Delta \chi ^2$ functions for $m_{\tilde{\chi }^0_{1}}$ (in the left panel) and $m_{\tilde{\chi }^\pm _{1}}$ (in the right panel) in the NUHM2, the NUHM1 and the CMSSM. The $\Delta \chi ^2$ functions for $m_{\tilde{\chi }^0_{1}}$ are quite similar at low masses, being largely driven by the ATLAS jets + ${/\!\!E}_T$ constraint, and we find that $m_{\tilde{\chi }^0_{1}} \gtrsim 250 \,\, \mathrm {GeV}$ at the 95 % CL. The $\Delta \chi ^2$ function in the NUHM2 (solid line) then has a shallow minimum for $m_{\tilde{\chi }^0_{1}} \in (600, 1000) \,\, \mathrm {GeV}$, with a best-fit value ${\sim } 760~\,\, \mathrm {GeV}$. As already mentioned, the NUHM2 best-fit point is in the stau coannihilation region, with $m_{\tilde{\tau }_1} - m_{\tilde{\chi }^0_{1}} \sim 18~\,\, \mathrm {GeV}$ and the other sleptons slightly heavier, as also seen in Fig. 2. In the case of $m_{\tilde{\chi }^\pm _{1}}$, the NUHM2 $\Delta \chi ^2$ function has a 95 % CL lower bound ${\gtrsim } 500 \,\, \mathrm {GeV}$ and a shallow minimum for $m_{\tilde{\chi }^\pm _{1}} \in (1000, 1500) \,\, \mathrm {GeV}$ and a best-fit value ${\sim } 1430~\,\, \mathrm {GeV}$ as also seen in Fig. 2. The extra degree of freedom in the NUHM2 compared to the NUHM1 does not relax significantly the lower bounds on the $\tilde{\chi }^\pm _{1}$ and $\tilde{\chi }^0_{1}$ masses.

**Fig. 12:**
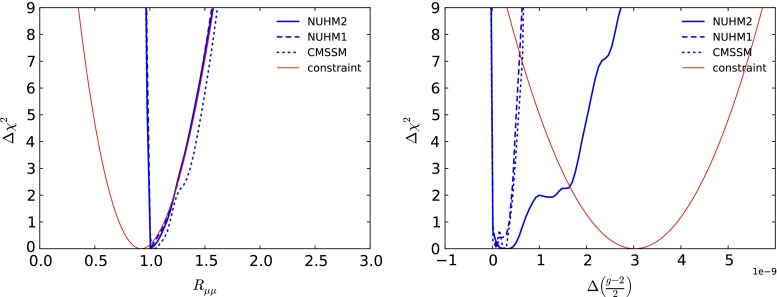
As in Fig. 8, for $R_{\mu \mu }$ (*left panel*) and for $\Delta \left( \frac{g-2}{2} \right)$ (*right panel*). In each panel, we display separately as a *red line* the contribution of that individual observable to the global $\chi ^2$ functions

The left panel of Fig. 12 displays the $\Delta \chi ^2$ functions for $R_{\mu \mu }$ (defined here as $BR(B_s \rightarrow \mu ^+\mu ^-)/BR(B_s \rightarrow \mu ^+\mu ^-)_\mathrm{SM}$) in the NUHM2, NUHM1 and CMSSM. We see that they are almost identical, and that all three models allow no scope for $R_{\mu \mu }$ to fall significantly below the SM value within the 95 % confidence level range. For $R_{\mu \mu }$ above the Standard Model value, the $\Delta \chi ^2$ functions all rise in the same way as the contribution from the experimental constraint on $R_{\mu \mu }$ (red line), implying that the other constraints do not impose significant constraints on $R_{\mu \mu }$ above the Standard Model value. The fact that the CMSSM appears to have slightly larger freedom for $R_{\mu \mu }$ is related to the fact the total $\chi ^2$ is larger than in the other models. Shifting the CMSSM curve in the right panel of Fig. 12 to account for that difference, the CMSSM region would be fully contained in the NUHM1,2 regions, as expected because of the stronger restrictions in the CMSSM.

### The anomalous magnetic moment of the muon

The right panel of Fig. 12 displays the $\Delta \chi ^2$ functions for the difference from the SM: $\Delta \left( \frac{g-2}{2} \right)$ in the NUHM2, NUHM1 and CMSSM, as blue solid, dashed and dotted lines, respectively. Also shown, as a solid red line, is the $(g-2)_\mu$ contribution to the $\chi ^2$ function. As is well known, the other constraints, principally those from the LHC, do not allow a large SUSY contribution to $(g-2)_\mu$ within the NUHM1 (dashed line) or the CMSSM (dotted line). We find that in the NUHM2 the most important role is played by the LHC $M_h$ measurement. As we also see in the right panel of Fig. 12, there is significantly more flexibility in the NUHM2 contribution to $(g-2)_\mu$ (solid line). However, even in this case the model is unable to reduce the discrepancy between the theoretical prediction and the central experimental value much below the $\Delta \chi ^2 \sim 9$ level. We find the $(g-2)_\mu$ contributions to the global $\chi ^2$ to be 9.2 (10.5) (8.8) in the CMSSM (NUHM1) (NUHM2). A reduction of the minimum value of the global $\chi ^2$ function w.r.t. the SM [19] is found at the level of $\Delta \chi ^2 \sim 4.0$, with a best-fit value of $\Delta \left( \frac{g-2}{2} \right) = 3.4 \times 10^{-10}$. Comparing with the NUHM1 (best-fit value $\Delta \left( \frac{g-2}{2} \right) = 1.0 \times 10^{-10}$), we find a reduction in the $(g-2)_\mu$ contribution to the global $\chi ^2$ function at the best-fit point by ${\sim } 1.6$, which is largely compensated by a net increase in the contributions of other observables, including the electroweak precision measurements. The best-fit value in the CMSSM is $\Delta \left( \frac{g-2}{2} \right) = 2.8 \times 10^{-10}$, with a total $\chi ^2$ higher than in the NUHM2 by 2.5. As seen in Fig. 11, in the low-mass regions the $\Delta \chi ^2$ functions for $m_{\tilde{\chi }^0_{1}}$ (in the left panel) and $m_{\tilde{\chi }^\pm _{1}}$ (in the right panel) in the NUHM2, the NUHM1 and the CMSSM are not very different. Going to lower mass, as would be needed for a further reduction in the $(g-2)_\mu$ discrepancy, is strongly penalised by the direct LHC searches for sparticles.

### Direct dark-matter detection

**Fig. 13:**
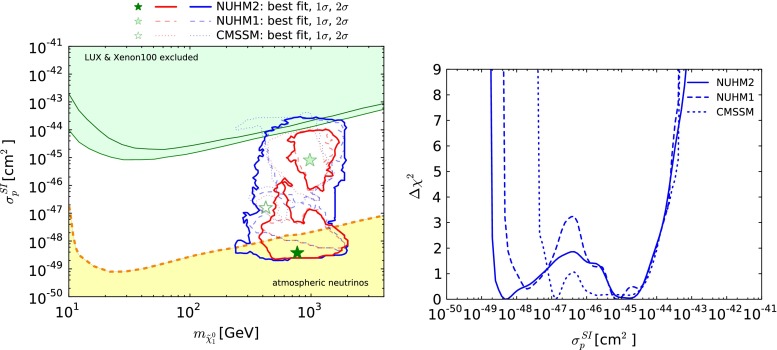
*Left panel* The $(m_{\tilde{\chi }^0_{1}}, \sigma ^\mathrm{SI}_p)$ plane in the NUHM2, with results in the CMSSM and NUHM1 shown for comparison. The *star* and *contours* have the same significations as in Fig. 1. Also shown are the 90 % CL upper limits on $\sigma ^\mathrm{SI}_p$ from the XENON100 [83] and LUX [84] experiments (*green* and *black lines*, respectively), and the calculated atmospheric neutrino background level from [169] (*orange dashed line*). *Right panel* The $\Delta \chi ^2$ functions for $\sigma ^\mathrm{SI}_p$ in the CMSSM, NUHM1 and NUHM2

The left panel of Fig. 13 displays the $(m_{\tilde{\chi }^0_{1}},\sigma ^\mathrm{SI}_p)$ plane, where $\sigma ^\mathrm{SI}_p$ is the spin-independent LSP-proton scattering cross section, including the best-fit points and the 68 and 95 % CL contours in the NUHM2, NUHM1 and CMSSM. Our computation of $\sigma ^\mathrm{SI}_p$ follows the procedure described in [19], and we have once again adopted for the $\pi$-nucleon $\sigma$ term the value $\Sigma _{\pi N} = 50 \pm 7$ MeV. In addition to the model results, we also display the 90 % CL upper limits on $\sigma ^\mathrm{SI}_p$ given by the XENON100 and LUX experiments [83, 84], and the level of the atmospheric neutrino background [169]. As we see in the right panel of Fig. 13, in the CMSSM the $\Delta \chi ^2$ function is relatively flat for $10^{-47}$ cm$^2 \lesssim$ $\sigma ^\mathrm{SI}_p$ $\lesssim 10^{-45}$ cm$^2$. On the other hand, in the case of the NUHM1, values of $\sigma ^\mathrm{SI}_p$ $\sim 10^{-48}$ cm$^2$ are only slightly disfavoured relative to the best-fit value of $\sigma ^\mathrm{SI}_p$ $\sim 10^{-45}$ cm$^2$, with intermediate values somewhat disfavoured. The low- and high-$\sigma ^\mathrm{SI}_p$ NUHM2 points with lowest $\chi ^2$ are in stau coannihilation regions, accompanied by $\tilde{\chi }^\pm _{1}$ coannihilation in the high-$\sigma ^\mathrm{SI}_p$ case, whereas the lowest-$\chi ^2$ points with intermediate values of $\sigma ^\mathrm{SI}_p$ are in $H/A$ funnel regions. The main differences in $\chi ^2$ between the high- and intermediate-$\sigma ^\mathrm{SI}_p$ points are due to $(g-2)_\mu$, and the largest differences in $\chi ^2$ between the low- and intermediate-$\sigma ^\mathrm{SI}_p$ points are due to $A_\mathrm{FB}(b)$. In the case of the NUHM2, values of $\sigma ^\mathrm{SI}_p$ $\sim 4 \times 10^{-49}$ cm$^2$, within the range where the atmospheric neutrino background dominates, are slightly favoured relative to the range $\sigma ^\mathrm{SI}_p\ \sim 10^{-45}$ cm$^2$. In all the three models, the steep rise in the $\Delta \chi ^2$ function at low values of $\sigma ^\mathrm{SI}_p$ is due to the contribution from Higgs exchange via the small Higgsino component in the $\tilde{\chi }^0_{1}$.

## Summary and conclusions

In this paper we have presented the results of a frequentist global fit of the NUHM2 model. Previous analyses of the CMSSM and NUHM1 models [19] have shown those models to be very constrained by available data. One might have wondered whether the extra degrees of freedom in the Higgs sector in the NUHM2 scenario would alleviate this tension, but we found that this was not the case.

Our fit employed ${\sim } 4 \times 10^8$ points in the NUHM2 parameter space, and we paid particular attention to the part of the NUHM2 parameter space where $m_0^2 < 0$. Applying the ATLAS constraints on jets + ${/\!\!E}_T$ to the NUHM1,2 (and especially to $m_0^2 < 0$) required an extrapolation from the published results, which we previously validated for 7 TeV limits using an implementation of the Delphes collider detector simulation code set to emulate the ATLAS detector.

The minimum value of $\chi ^2/\mathrm{dof}$ was $32.5/21$, to be compared with the values $\chi ^2/\mathrm{dof} \sim 32.7/22$ and $35.0/23$ found in our previous analyses of the NUHM1 and CMSSM, respectively. We found that ranges of $m_{H_u}^2 < m_{H_d}^2 < m_0^2 < 0$ are favoured. We find similar tension between $(g-2)_\mu$ and the LHC Higgs and jets + ${/\!\!E}_T$ constraints in the NUHM2 as in the NUHM1 and CMSSM. The best-fit values of $m_{\tilde{g}}$ and $m_{\tilde{q}_R}$ in the NUHM2 are ${\sim } 3 \,\, \mathrm {TeV}$, with $\chi ^2$ functions that are quite flat for masses ${\gtrsim } 2 \,\, \mathrm {TeV}$. The freedom effectively to vary $\mu$ and $M_A$ in the NUHM2 does not suffice to provide a better fit to $(g-2)_\mu$  and suggests that if this anomaly persists then some non-universality among the SUSY-breaking scalar masses may be required.

On the one hand, it is encouraging that the results of this NUHM2 analysis are relatively similar to those found previously for the NUHM1 and the CMSSM, suggesting that the type of frequentist analysis presented here is robust with respect to simple expansions of the CMSSM parameter space. On the other hand, this analysis suggests that it would be interesting to study models in which the GUT universality assumptions are further relaxed, with a corresponding increase in the number of parameters. Such models may offer the prospect of a significant reduction in $\chi ^2$ if they can relax the tension between $(g-2)_\mu$ and the LHC constraints. Similarly, models based on a phenomenological definition of low-energy soft supersymmetry-breaking parameters, variants of the pMSSM [170–177], may also ameliorate the tension. This may offer another path of extension beyond the well-studied CMSSM, NUHM1 and NUHM2 scenarios.

## Footnotes

1 For a previous scan of the NUHM2 parameter space using only 7-TeV LHC data, which did not make a global fit; see [42].

2 See also the similar CMS result [11], which we do not use here, as the published description of the ATLAS analysis enables us to model better its contribution to the global $\chi ^2$ function.

3 The number of $1.5 \,\, \mathrm {GeV}$ is treated as a Gaussian uncertainty at the 68 % CL. Consequently, an uncertainty of $3 \,\, \mathrm {GeV}$ is assigned at the 95 % CL, in agreement with the analyses in [97–101].

4 As in [19], we do not include constraints from the Higgs signal strength measurements. These are not yet sufficiently accurate to constrain our results. The rate predictions in the favoured regions of the NUHM2, NUHM1 and CMSSM are dominated by large values of $M_A$ in the TeV range, where the MSSM decouples to the SM limit [135–138]. Consequently, the rates predicted for the light Higgs are SM-like, despite the additional freedom for $M_A$ in the NUHM2 and NUHM1.

5 Searches with leptons and/or $b$ quarks are important at large $m_0$, but they extend only to values of $m_{1/2}$ that are disfavoured already by other constraints.

6 In the NUHM1, the flexibility to allow $m^2_{H_d} = m^2_{H_u} < 0$ with a different value from $m^2_0$ can also affect the running to ensure a neutralino LSP when $m^2_0 < 0$, but only in a restricted region when $m_{1/2}$ is large enough.

7 We do not show results for $\tan \beta > 60$ where the RGE results are less reliable.

8 However, the exact locations of the CL contours near the best-fit point in the right panel of Fig. 4 are subject to our sampling restrictions. For comparison, these plots also show the corresponding contours for the NUHM1, which are identical in the two panels because $m_{H_u}^2 = m_{H_d}^2$ in the NUHM1.

9 We recall that we use the same convention for the sign of $A_0$ as in [19, 92], which is opposite to the convention used in, e.g., SoftSUSY.

10 The best-fit value of $m_{H_d}^2$ lies slightly outside the nominal range of our scan, but within a region that is sampled with ${\sim } 50$ % of the density within the range. We have verified using MINUIT that although the global $\chi ^2$ function is quite flat, it has a well-defined minimum at this point.

11 The kink in the $\chi ^2$ function is due to the stop coannihilation mechanism for reducing $\Omega _\chi h^2$ becoming inoperative. Similar effects are seen in other likelihood functions.

## References

[1] L. Maiani, in *Recent Developments in Gauge Theories, Proceedings of the Nato Advanced Study Institute, Cargese, 1979*, ed. by G. ’t Hooft et al. Proceedings of the 1979 Gif-sur-Yvette Summer School On Particle Physics, G. ’t Hooft (Plenum Press, New York, 1980)

[2] Witten E (1981). Phys. Lett. B.

[3] Ellis J, Kelley S, Nanopoulos DV (1990). Phys. Lett. B.

[4] Ellis J, Kelley S, Nanopoulos DV (1991). Phys. Lett. B.

[5] Amaldi U, de Boer W, Furstenau H (1991). Phys. Lett. B.

[6] Langacker P, Luo M-X (1991). Phys. Rev. D.

[7] Giunti C, Kim CW, Lee UW (1991). Mod. Phys. Lett. A.

[8] Goldberg H (1983). Phys. Rev. Lett..

[9] Ellis J, Hagelin J, Nanopoulos D, Olive K, Srednicki M (1984). Nucl. Phys. B.

[10] G. Aad et al. (ATLAS Collaboration), arXiv:1405.7875 [hep-ex]

[11] S. Chatrchyan et al. (CMS Collaboration), JHEP **1406**, 055 (2014). arXiv:1402.4770 [hep-ex]

[12] G. Aad et al. (ATLAS Collaboration), Phys. Lett. B **716**, 1 (2012). arXiv:1207.7214 [hep-ex]

[13] S. Chatrchyan et al. (CMS Collaboration), Phys. Lett. B **716**, 30 (2012). arXiv:1207.7235 [hep-ex]

[14] R. Aaij et al. (LHCb Collaboration), Phys. Rev. Lett. **111**, 101805 (2013). arXiv:1307.5024 [hep-ex]

[15] S. Chatrchyan et al. (CMS Collaboration), Phys. Rev. Lett. **111**, 101804 (2013). arXiv:1307.5025 [hep-ex] 10.1103/PhysRevLett.111.101804 25166654

[16] R. Aaij et al. (LHCb and CMS Collaborations), LHCb-CONF-2013-012, CMS PAS BPH-13-007

[17] Nilles HP (1984). Phys. Rep..

[18] Haber HE, Kane GL (1985). Phys. Rep..

[19] Buchmueller O (2014). Eur. Phys. J. C.

[20] Li T, Maxin JA, Nanopoulos DV, Walker JW (2012). Phys. Lett. B.

[21] Dolan MJ (2011). JHEP.

[22] Heinemeyer S, Stal O, Weiglein G (2012). Phys. Lett. B.

[23] Arbey A, Battaglia M, Djouadi A, Mahmoudi F, Quevillon J (2012). Phys. Lett. B.

[24] Draper P, Meade P, Reece M, Shih D (2012). Phys. Rev. D.

[25] Akula S, Altunkaynak B, Feldman D, Nath P, Peim G (2012). Phys. Rev. D.

[26] Kadastik M, Kannike K, Racioppi A, Raidal M (2012). JHEP.

[27] Strege C (2012). JCAP.

[28] Cao J, Heng Z, Li D, Yang JM (2012). Phys. Lett. B.

[29] Aparicio L, Cerdeno DG, Ibanez LE (2012). JHEP.

[30] Baer H, Barger V, Mustafayev A (2012). JHEP.

[31] Bechtle P (2012). JHEP.

[32] Bechtle P (2013). Eur. Phys. J. C.

[33] Ghosh D, Guchait M, Raychaudhuri S, Sengupta D (2012). Phys. Rev. D.

[34] Fowlie A, Kazana M, Kowalska K, Munir S, Roszkowski L, Sessolo EM, Trojanowski S, Tsai Y-LS (2012). Phys. Rev. D.

[35] K. Kowalska et al. (BayesFITS Group Collaboration), Phys. Rev. D **87**, 115010 (2013). arXiv:1211.1693 [hep-ph]

[36] Strege C, Bertone G, Feroz F, Fornasa M, Ruiz de Austri R, Trotta R (2013). JCAP.

[37] Cabrera ME, Casas JA, de Austri RR (2013). JHEP.

[38] Cohen T, Wacker JG (2013). JHEP.

[39] Henrot-Versillé S (2014). Phys. Rev. D.

[40] P. Bechtle et al., PoS **EPS-HEP2013**, 31 (2013). arXiv:1310.3045 [hep-ph]

[41] L. Roszkowski, E.M. Sessolo, A.J. Williams, arXiv:1405.4289 [hep-ph]

[42] H. Baer, V. Barger, A. Mustafayev, arXiv:1112.3017 [hep-ph]

[43] Drees M, Nojiri MM (1993). Phys. Rev. D.

[44] Baer H, Brhlik M (1996). Phys. Rev. D.

[45] Baer H, Brhlik M (1998). Phys. Rev. D.

[46] Baer H, Brhlik M, Diaz MA, Ferrandis J, Mercadante P, Quintana P, Tata X (2001). Phys. Rev. D.

[47] Ellis JR, Falk T, Ganis G, Olive KA, Srednicki M (2001). Phys. Lett. B.

[48] Kane GL, Kolda CF, Roszkowski L, Wells JD (1994). Phys. Rev. D.

[49] Ellis JR, Falk T, Olive KA, Schmitt M (1996). Phys. Lett. B.

[50] Ellis JR, Falk T, Olive KA, Schmitt M (1997). Phys. Lett. B.

[51] Ellis JR, Falk T, Ganis G, Olive KA, Schmitt M (1998). Phys. Rev. D.

[52] Barger VD, Kao C (1998). Phys. Rev. D.

[53] Ellis JR, Falk T, Ganis G, Olive KA (2000). Phys. Rev. D.

[54] Roszkowski L, Ruiz de Austri R, Nihei T (2001). JHEP.

[55] Djouadi A, Drees M, Kneur JL (2001). JHEP.

[56] Chattopadhyay U, Corsetti A, Nath P (2002). Phys. Rev. D.

[57] Ellis JR, Olive KA, Santoso Y (2002). New J. Phys..

[58] Baer H, Balazs C, Belyaev A, Mizukoshi JK, Tata X, Wang Y (2002). JHEP.

[59] R. Arnowitt, B. Dutta, arXiv:hep-ph/0211417

[60] AbdusSalam SS (2011). Eur. Phys. J. C.

[61] Baer H, Mustafayev A, Profumo S, Belyaev A, Tata X (2005). Phys. Rev. D.

[62] Baer H, Mustafayev A, Profumo S, Belyaev A, Tata X (2005). JHEP.

[63] Ellis JR, Olive KA, Sandick P (2008). Phys. Rev. D.

[64] Ellis J, Luo F, Olive KA, Sandick P (2013). Eur. Phys. J. C.

[65] G. Bennett et al. (The Muon g-2 Collaboration), Phys. Rev. Lett. **92**, 161802 (2004). arXiv:hep-ex/0401008

[66] G. Bennett et al. (The Muon g-2 Collaboration), Phys. Rev. D **73**, 072003 (2006). arXiv:hep-ex/0602035

[67] Stockinger D (2007). J. Phys. G.

[68] Miller J, de Rafael E, Roberts B (2007). Rep. Prog. Phys..

[69] J. Prades, E. de Rafael, A. Vainshtein, arXiv:0901.0306 [hep-ph]

[70] Jegerlehner F, Nyffeler A (2009). Phys. Rep..

[71] Davier M, Hoecker A, Malaescu B, Yuan CZ, Zhang Z (2010). Eur. Phys. J. C.

[72] J. Prades, Acta Phys. Polon. Supp. **3**, 75 (2010). arXiv:0909.2546 [hep-ph]

[73] T. Teubner, K. Hagiwara, R. Liao, A.D. Martin, D. Nomura, arXiv:1001.5401 [hep-ph]

[74] Davier M, Hoecker A, Malaescu B, Zhang Z (2011). Eur. Phys. J. C.

[75] Jegerlehner F, Szafron R (2011). Eur. Phys. J. C.

[76] Benayoun M, David P, DelBuono L, Jegerlehner F (2013). Eur. Phys. J. C.

[77] Buchmueller O (2012). Eur. Phys. J. C.

[78] Ellis J, Olive K, Santoso Y (2002). Phys. Lett. B.

[79] Ellis JR, Falk T, Olive KA, Santoso Y (2003). Nucl. Phys. B.

[80] Feroz F, Hobson MP, Bridges M (2009). Mon. Not. R. Astron. Soc..

[81] The Heavy Flavor Averaging Group, D. Asner et al., arXiv:1010.1589 [hep-ex], with updates available at http://www.slac.stanford.edu/xorg/hfag/osc/end_2009

[82] LEP Electroweak Working Group, http://lepewwg.web.cern.ch/LEPEWWG/

[83] E. Aprile et al. (XENON100 Collaboration), Phys. Rev. Lett. **107**, 131302 (2011). arXiv:1104.2549 [astro-ph.CO] 10.1103/PhysRevLett.107.131302 22026838

[84] D.S. Akerib et al. (LUX Collaboration), Phys. Rev. Lett. **112**, 091303 (2014). arXiv:1310.8214 [astro-ph.CO]

[85] Feng JL, Rajaraman A, Smith BT (2006). Phys. Rev. D.

[86] Rajaraman A, Smith BT (2007). Phys. Rev. D.

[87] O. Lebedev, H.P. Nilles, M. Ratz, hep-ph/0511320

[88] Craig N, Knapen S, Shih D, Zhao Y (2013). JHEP.

[89] Falk T, Olive KA, Roszkowski L, Srednicki M (1996). Phys. Lett. B.

[90] Falk T, Olive KA, Roszkowski L, Singh A, Srednicki M (1997). Phys. Lett. B.

[91] Ellis JR, Giedt J, Lebedev O, Olive K, Srednicki M (2008). Phys. Rev. D.

[92] Buchmueller O (2012). Eur. Phys. J. C.

[93] http://gfitter.desy.de/Figures/Standard_Model/2013_05_29_ShowFullFitTable_large.gif. Accessed 18 Aug 2014

[94] ALEPH, CDF, D0, DELPHI, L3, OPAL, SLD Collaborations, the LEP Electroweak Working Group, the Tevatron Electroweak Working Group and the SLD electroweak and heavy flavour groups, arXiv:1012.2367 [hep-ex], as updated in July 2011 on http://lepewwg.web.cern.ch/LEPEWWG/plots/summer2011/

[95] Heinemeyer S (2006). JHEP.

[96] Heinemeyer S, Hollik W, Weber AM, Weiglein G (2008). JHEP.

[97] Degrassi G, Heinemeyer S, Hollik W, Slavich P, Weiglein G (2003). Eur. Phys. J. C.

[98] Heinemeyer S, Hollik W, Weiglein G (1999). Eur. Phys. J. C.

[99] Heinemeyer S, Hollik W, Weiglein G (2000). Comput. Phys. Commun..

[100] Frank M (2007). JHEP.

[101] See http://www.feynhiggs.de. Accessed 18 Aug 2014

[102] Misiak M (2007). Phys. Rev. Lett..

[103] Ciuchini M, Degrassi G, Gambino P, Giudice GF (1998). Nucl. Phys. B.

[104] Degrassi G, Gambino P, Giudice GF (2000). JHEP.

[105] Carena MS, Garcia D, Nierste U, Wagner CEM (2001). Phys. Lett. B.

[106] D’Ambrosio G, Giudice GF, Isidori G, Strumia A (2002). Nucl. Phys. B.

[107] C. Bobeth, M. Gorbahn, T. Hermann, M. Misiak, E. Stamou, M. Steinhauser, arXiv:1311.0903 [hep-ph] 10.1103/PhysRevLett.112.101801 24679279

[108] T. Hermann, M. Misiak, M. Steinhauser, arXiv:1311.1347 [hep-ph]

[109] C. Bobeth, M. Gorbahn, E. Stamou, arXiv:1311.1348 [hep-ph]

[110] Buras AJ (2003). Phys. Lett. B.

[111] Isidori G, Straub DM (2012). Eur. Phys. J. C.

[112] Isidori G, Paradisi P (2006). Phys. Lett. B.

[113] G. Isidori, F. Mescia, P. Paradisi, D. Temes, Phys. Rev. D **75**, 115019 (2007). arXiv:hep-ph/0703035, and references therein

[114] K.A. Olive et al. (Particle Data Group Collaboration), Chin. Phys. C **38**, 090001 (2014)

[115] Bobeth C, Buras AJ, Ewerth T (2005). Nucl. Phys. B.

[116] M. Antonelli et al. (FlaviaNet Working Group on Kaon Decays), arXiv:0801.1817 [hep-ph]

[117] Buras AJ, Gambino P, Gorbahn M, Jager S, Silvestrini L (2001). Nucl. Phys. B.

[118] A.V. Artamonov et al. (The E949 Collaboration), arXiv:0808.2459 [hep-ex]

[119] R. Aaij et al. (LHCb Collaboration), New J. Phys. **15**, 053021 (2013). arXiv:1304.4741 [hep-ex]

[120] Belanger G, Boudjema F, Pukhov A, Semenov A (2007). Comput. Phys. Commun..

[121] Belanger G, Boudjema F, Pukhov A, Semenov A (2002). Comput. Phys. Commun..

[122] Belanger G, Boudjema F, Pukhov A, Semenov A (2006). Comput. Phys. Commun..

[123] See Table 5 of P.A.R. Ade et al. (Planck Collaboration), Astron. Astrophys. (2014). arXiv:1303.5076 [astro-ph.CO]

[124] ATLAS Collaboration, https://cds.cern.ch/record/1744694/files/ATLAS-CONF-2014-049

[125] See also V. Khachatryan et al. (CMS Collaboration), arXiv:1408.3316 [hep-ex]

[126] For more information and updates, please see http://cern.ch/mastercode/. Accessed 18 Aug 2014

[127] Allanach BC (2002). Comput. Phys. Commun..

[128] Hahn T, Heinemeyer S, Hollik W, Rzehak H, Weiglein G (2014). Phys. Rev. Lett..

[129] Mahmoudi F (2008). Comput. Phys. Commun..

[130] Mahmoudi F (2009). Comput. Phys. Commun..

[131] Eriksson D, Mahmoudi F, Stal O (2008). JHEP.

[132] Information about this code is available from K.A. Olive: it contains important contributions from T. Falk, A. Ferstl, G. Ganis, A. Mustafayev, J. McDonald, F. Luo, K.A. Olive, P. Sandick, Y. Santoso, V. Spanos, M. Srednicki

[133] Skands P (2004). JHEP.

[134] Allanach B (2009). Comput. Phys. Commun..

[135] Dobado A, Herrero MJ, Penaranda S (2000). Eur. Phys. J. C.

[136] Gunion J, Haber H (1993). Phys. Rev. D.

[137] Haber H, Nir Y (1993). Phys. Lett. B.

[138] H. Haber, arXiv:hep-ph/9505240

[139] Buchmueller O (2014). Eur. Phys. J. C.

[140] For a description of Delphes, written by S. Ovyn and X. Rouby, see http://www.fynu.ucl.ac.be/users/s.ovyn/Delphes/index.html

[141] V. Khachatryan et al. (CMS Collaboration), Phys. Lett. B **698**, 196 (2011). arXiv:1101.1628 [hep-ex]

[142] CMS Collaboration, http://cds.cern.ch/record/1343076. See, in particular, the Figure https://twiki.cern.ch/twiki/pub/CMSPublic/PhysicsResultsSUS10005/CMSSM_m0_m12_Comp from this paper

[143] Martin SP, Vaughn MT (1994). Phys. Rev. D.

[144] K. Inoue, A. Kakuto, H. Komatsu, S. Takeshita, Prog. Theor. Phys. **68**, 927 (1982) [Erratum-ibid. **70**, 330 (1983)]

[145] Falk T (1999). Phys. Lett. B.

[146] Feng JL, Matchev KT, Moroi T (2000). Phys. Rev. Lett..

[147] Feng JL, Matchev KT, Moroi T (2000). Phys. Rev. D.

[148] Feng JL, Matchev KT, Wilczek F (2000). Phys. Lett. B.

[149] Ellis J, Falk T, Olive KA (1998). Phys. Lett. B.

[150] J. Ellis, T. Falk, K.A. Olive, M. Srednicki, Astropart. Phys. **13**, 181 (2000). arXiv:hep-ph/9905481 [Erratum-ibid. **15**, 413 (2001)]

[151] Arnowitt R, Dutta B, Santoso Y (2001). Nucl. Phys. B.

[152] Gómez ME, Lazarides G, Pallis C (2000). Phys. Rev. D.

[153] M.E. Gómez, G. Lazarides, C. Pallis, Phys. Lett. B **487**, 313 (2000). arXiv:hep-ph/0004028

[154] M.E. Gómez, G. Lazarides, C. Pallis, Nucl. Phys. B **B638**, 165 (2002). arXiv:hep-ph/0203131

[155] Nihei T, Roszkowski L, Ruiz de Austri R (2002). JHEP.

[156] Citron M, Ellis J, Luo F, Marrouche J, Olive KA, de Vries KJ (2013). Phys. Rev. D.

[157] Edsjo J, Schelke M, Ullio P, Gondolo P (2003). JCAP.

[158] Mizuta S, Yamaguchi M (1993). Phys. Lett. B.

[159] Edsjo J, Gondolo P (1997). Phys. Rev. D.

[160] Baer H, Balazs C, Belyaev A (2002). JHEP.

[161] A. Birkedal-Hansen, E.h. Jeong, JHEP **0302**, 047 (2003). hep-ph/0210041

[162] Boehm C, Djouadi A, Drees M (2000). Phys. Rev. D.

[163] Ellis JR, Olive KA, Santoso Y (2003). Astropart. Phys..

[164] Diaz-Cruz JL, Ellis JR, Olive KA, Santoso Y (2007). JHEP.

[165] Gogoladze I, Raza S, Shafi Q (2012). Phys. Lett. B.

[166] Ajaib MA, Li T, Shafi Q (2012). Phys. Rev. D.

[167] J. Ellis, K.A. Olive, J. Zheng, arXiv:1404.5571 [hep-ph]

[168] Camargo-Molina JE, O’Leary B, Porod W, Staub F (2013). Eur. Phys. J. C.

[169] P. Cushman et al., arXiv:1310.8327 [hep-ex]

[170] See, for example, C.F. Berger, J.S. Gainer, J.L. Hewett, T.G. Rizzo, JHEP **0902**, 023 (2009). arXiv:0812.0980 [hep-ph]

[171] AbdusSalam SS, Allanach BC, Quevedo F, Feroz F, Hobson M (2010). Phys. Rev. D.

[172] Conley JA, Gainer JS, Hewett JL, Le MP, Rizzo TG (2011). Eur. Phys. J. C.

[173] J.A. Conley, J.S. Gainer, J.L. Hewett, M.P. Le, T.G. Rizzo, arXiv:1103.1697 [hep-ph]

[174] Sekmen S, Kraml S, Lykken J, Moortgat F, Padhi S, Pape L, Pierini M, Prosper HB (2012). JHEP.

[175] Arbey A, Battaglia M, Mahmoudi F (2012). Eur. Phys. J. C.

[176] Arbey A, Battaglia M, Djouadi A, Mahmoudi F (2013). Phys. Lett. B.

[177] M.W. Cahill-Rowley, J.L. Hewett, A. Ismail, T.G. Rizzo, Phys. Rev. D **88**, 3, 035002 (2013). arXiv:1211.1981 [hep-ph]

